# OCT-Angiography Findings in Patients with Amblyopia: Comparison between Healthy Controls, Treatment-Responsive, and Treatment-Unresponsive Amblyopic Patients

**DOI:** 10.3390/diagnostics11101751

**Published:** 2021-09-24

**Authors:** Annabella Salerni, Gloria Gambini, Chiara Fedeli, Ludovica Paris, Emanuele Crincoli, Gustavo Savino, Maria Cristina Savastano, Daniela Bacherini, Umberto De Vico, Clara Rizzo, Raphael Killian, Stanislao Rizzo

**Affiliations:** 1 Ophthalmology Unit, “Fondazione Policlinico Universitario A. Gemelli IRCCS”, 00168 Rome, Italy; annabella.salerni@policlinicogemelli.it (A.S.); chiara.fedeli@policlinicogemelli.it (C.F.); ludovica.paris@libero.it (L.P.); emanuelecrincoli1@gmail.com (E.C.); gustavo.savino@unicatt.it (G.S.); mariacristina.savastano@gmail.com (M.C.S.); umbertodevico@gmail.com (U.D.V.); stanislao.rizzo@gmail.com (S.R.); 2 Ophthalmology Unit, Catholic University “Sacro Cuore”, 00168 Rome, Italy; 3 Department of Surgery and Translational Medicine, AOU Careggi, University of Florence, 50139 Florence, Italy; daniela.bacherini@gmail.com; 4 Ophthalmology, Department of Surgical, Medical and Molecular Pathology and Critical Care Medicine, University of Pisa, 56126 Pisa, Italy; clararizzo2@gmail.com; 5 Ophthalmology Unit, University of Verona, 37134 Verona, Italy; raphaelkilian8@yahoo.it; 6 Istituto di Neuroscienze, Consiglio Nazionale delle Ricerche, 56124 Pisa, Italy

**Keywords:** amblyopia, optical coherence tomography angiography, pediatric ophthalmology, retina, innovative biotechnology, personalized medicine

## Abstract

There is no consensus on whether amblyopia affects the retinal vascular plexus and morphology. Previous studies focused on the differences between amblyopic patients and normal controls without evaluating amblyopic eyes after patching. To evaluate differences in the superficial vascular density of amblyopic eyes, normal eyes, and amblyopic eyes reaching normal BCVA after patch therapy, OCTA was used. All patients underwent a comprehensive ophthalmological examination, including visual acuity, refraction, ocular motility tests, and anterior and posterior segment examination. OCTA was performed by an expert physician using the Zeiss Cirrus 5000-HD-OCT Angioplex (Carl Zeiss, Meditec, Inc., Dublin, OH, USA). OCTA scans were performed using a 3 × 3 mm^2^ and 6 × 6 mm^2^ fovea-centered image setting. The mean outer macular vessel density in the previously amblyopic group was 19.15 ± 0.51%. This was statistically significantly higher than in both the amblyopic group (18.70 ± 1.14%) and the normal controls (18.18 ± 1.40%) (*p* = 0.014). The previously amblyopic group also significantly differed from both normal controls and amblyopic eyes with regards to the inner (*p* = 0.011), outer (*p* = 0.006), and full (*p* = 0.003) macular perfusion. Finally, linear regression analysis revealed that BCVA was linearly correlated to outer perfusion in amblyopic (*p* = 0.003) and ex amblyopic eyes (*p* < 0.001). Considering the cross-sectional nature of our study, from our results, we can only hypothesize a possible correlation between light stimulation and retinal vasculature development. However, further longitudinal studies are needed to support this hypothesis.

## 1. Introduction

Amblyopia is the most common cause of monocular and binocular vision loss in children and its population-based prevalence ranges from 0.5% to 3.5% [1,2,3,4]. Amblyopia is traditionally considered a cortical disease. The involvement of other structures in the visual pathway related to this disease is still to be determined [5,6]. Microscopic and functional abnormalities have been reported in the lateral geniculate nucleus, but the involvement of the retina is still debated [7,8,9]. With the advances in optical coherence tomography (OCT), several studies have shown structural abnormalities or modifications in the retina, the optic nerve, and the choroid of amblyopic eyes, compared to normal controls [10,11,12,13,14,15]. The results are often contradictory due to different confounding factors.

Nowadays, OCT angiography (OCTA) is becoming part of everyday clinical practice as a fundamental technique for the study of the retinal microvasculature. Because it is a dye-free technique and requires a short time for acquisition, this procedure is very suitable for examination of children’s eyes. OCTA allows for a stratified evaluation of the retinal microvasculature, which cannot be performed with conventional fluorescein angiography. Furthermore, it can quantify foveal avascular zone (FAZ) area, circularity, macular vessel density (VD), and perfusion (VP) [16]. Up to now, there is no consensus on whether amblyopia affects the retinal vascular plexus and the morphology of the foveal avascular zone. Most of the currently available studies focus on the differences between amblyopic patients and normal controls without evaluating amblyopic eyes that had achieved normal best corrected visual acuity (BCVA) after patching. Indeed, a post-treatment analysis could establish the reversibility of some of the foveal abnormalities that characterize amblyopic eyes. Particularly, post-treatment OCTA examination could clarify if the vascular abnormalities of amblyopic eyes are permanent or can regress.

The aim of our study was to evaluate differences in the superficial vascular density of three groups (i.e., amblyopic eyes, normal eyes, and amblyopic eyes reaching normal BCVA after patch therapy) using OCTA. Secondary outcomes were to observe the differences in three FAZ morphological features (i.e., area, perimeter, acircularity index) among the same three groups of patients.

## 2. Materials and Methods

The study was supported by Fondazione Policlinico A. Gemelli IRCSS Rome, Italy and designed by the investigators. It was approved by the Institutional Ethical Committee of Catholic University “Sacro Cuore”, Rome. The study was conducted in accordance with the tenets of the declaration of Helsinki and an informed consent was obtained from all participants and from the participants’ parents. All the authors reviewed the manuscript and vouch for the accuracy and completeness of the data and for the adherence of the study to the protocol. The study was designed as single-centered, observational, and cross-sectional. All patients’ data as well as patients’ investigations performed in this study were deposited in the REDCap system of Fondazione Policlinico Universitario A. Gemelli IRCCS Data Center, Rome, Italy [17].

Eyes in the amblyopic group were selected from pediatric patients receiving a diagnosis of amblyopia during a routine visit in the pediatric eye clinic. All patients included in this group had undergone anti-amblyopia therapy via the application of a patch on their best functioning eye but did not respond to the treatment. Amblyopia due to strabismus, anisometropia and meridional amblyopia were included. Amblyopia was defined as the reduction in best corrected visual acuity of one or both eyes that cannot be attributed exclusively to a structural abnormality of the eye. It is clinically defined as a difference in best corrected visual acuity of 2 or more lines of acuity between the eyes.

Eyes that underwent successful patching treatment were selected from patients that used to be amblyopic in one or both eyes and that achieved a BCVA of 20/20 after the treatment. Eyes with an initial diagnosis of strabismus, anisometropia, and meridional related amblyopia before beginning patch therapy were included.

For the normal control group, we selected normal pediatric patients who attended the eye clinic for a routine examination with a BCVA 20/20 and no evidence of ocular abnormalities.

Exclusion criteria for all groups were the presence of deprivation amblyopia, nystagmus, retinal disease, intraocular inflammation, and media opacity such as corneal disease or cataract. Patients with a history of prematurity, neurologic disease, or systemic conditions including diabetes, hypertension, cardiovascular disease, and renal disease were also excluded. In addition, patients with an inability to maintain retinal fixation on a specified target were excluded. OCTA scan quality inferior to 7/10 or with artifacts also led to rejection from analysis.

The anti-amblyopia treatment that was performed was occlusion therapy in the form of patching. Patching was performed by specifically occluding the sound eye in monolateral amblyopia and using alternate occlusion in bilateral amblyopia. The number of hours per day was established considering age of the patient and stage of amblyopia. For the amblyopic group, the average number of hours of patching per day was 4.3 h (in the range of 3–8 h) and for the previously amblyopic responsive group, 3.8 h (in the range of 2–8 h).

All patients underwent a comprehensive ophthalmological examination, including visual acuity, (Snellen BCVA was converted to logMAR for the analysis), refraction, ocular motility tests, and anterior and posterior segment examination (SL9900 Slit Lamp, CSO, Florence, Italy). OCTA was performed by an expert physician using the Zeiss Cirrus 5000-HD-OCT Angioplex (sw version 10.0, Carl Zeiss, Meditec, Inc., Dublin, OH, USA). OCT-A scans were performed using a 3 × 3 mm^2^ and 6 × 6 mm^2^ fovea-centered image setting.

**Figure 1:**
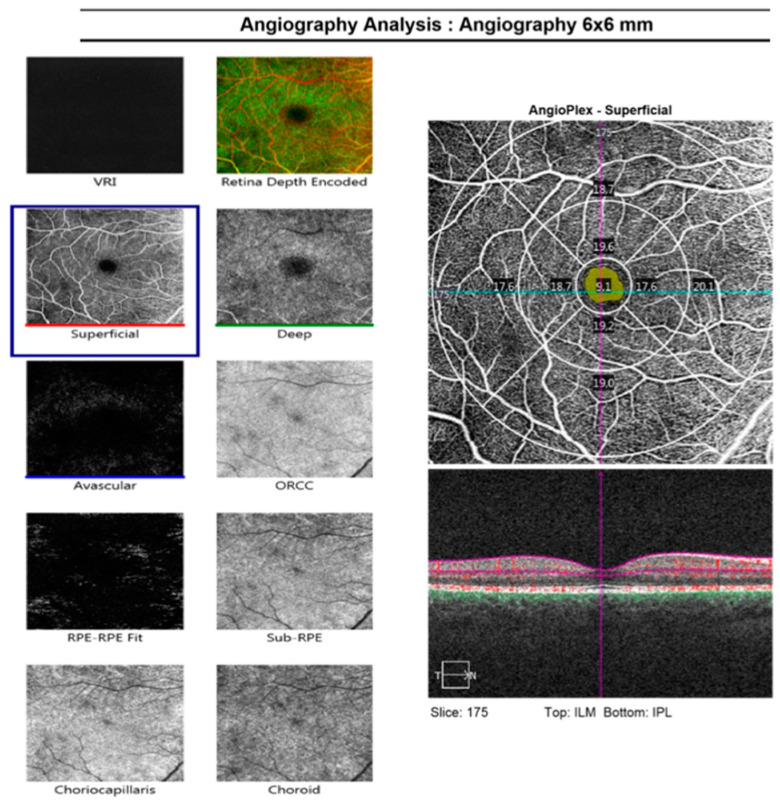
Sample OCT angiography of a previously amblyopic eye that had been treated by patching. Legend for macular regions mentioned in the main text is illustrated in the bottom right of the image.

The OCTA images were obtained during the final visit at the end of the treatment period. Two expert masked readers evaluated images of structural OCT and OCTA for quality control. All images with significant artifacts or poor quality were excluded from analysis (Figure 1).

The entire enface microvasculature was evaluated in the 6 × 6 mm^2^ area. Superficial retinal capillary plexus (SCP) was automatically segmented between the internal limiting membrane (ILM) and the outer boundary of the inner plexiform layer (IPL). FAZ area (mm^2^), perimeter (mm), and acircularity index (AI) were evaluated on the 3 × 3 mm^2^ scan. The FAZ parameters were measured in the slab corresponding to the SP using the automated software of the Zeiss Cirrus 5000-HD-OCT Angioplex (sw version 10.0, Carl Zeiss, Meditec, Inc., Dublin, OH, USA).

The primary endpoint was a difference in the VD between the three groups. The following parameters were chosen as secondary outcome measures: differences in FAZ area, FAZ perimeter, and FAZ acircularity between the three groups. Potential confounders taken into account included age, refractive error, and type of amblyopia.

## 3. Statistical Analysis

The sample size calculation was performed using G*power (3.1.9.7 software) by setting the desired power of the study to 20%, the alpha error to 5%, and a clinically significant difference of 5% in VD. Statistical analysis was conducted using SPSS software (IBM SPSS Statistics 26.0). With regards to the quantitative variables, normality of the distribution was evaluated using Shapiro–Wilk test. Preliminary evaluation was assessed with one-way ANOVA for quantitative variables and with Chi2 test or Fisher exact test with Bonferroni post-hoc correction for qualitative variables. Multivariate analysis of statistically significant quantitative variables between the three groups was performed using a two-way MANOVA with Bonferroni post-hoc test.The ability of a variable to differentiate among ex amblyopic eyes, amblyopic eyes, and normal eyes was evaluated by calculating the area under the receiver operating characteristic curve (AUC), with AUC = 0.5 being null (chance) and then increasing from 0.6 (poor) to 1 (excellent). Sensitivity and specificity at the various levels of the ROC curve were used to identify the most appropriate cut-off through minimization of the distance of the curve from the (0, 1) upper left vertex D = ((1 − SNS)·2 + (1 − SPC)·2)·0.5. Differences between the AUCs were tested for statistical significance with the method of Hanley and McNeil. Quantitative variables showing statistically significant differences between groups were tested for correlation with BCVA using multiple linear regression. The agreement between the two graders in manual measurements (FAZ area) was determined through Bland–Altman plot analysis.

## 4. Results

**Figure 2:**
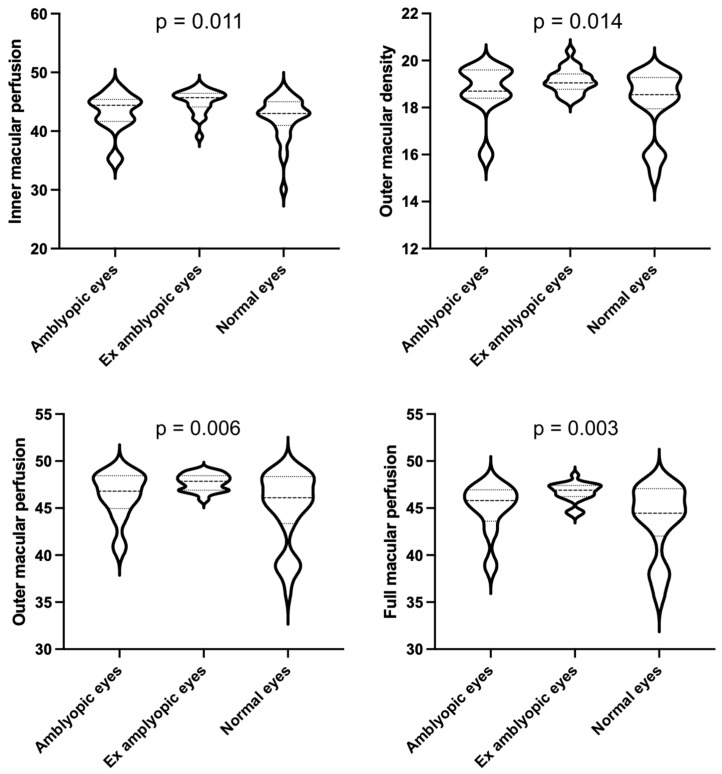
Violin plots illustrating OCT-A parameters statistically significantly differing in ex amblyopic eyes compared to amblyopic and normal eyes. All OCT-A parameters are expressed in percentage. *p* values are shown in the upper side of each graph.

**Figure 3:**
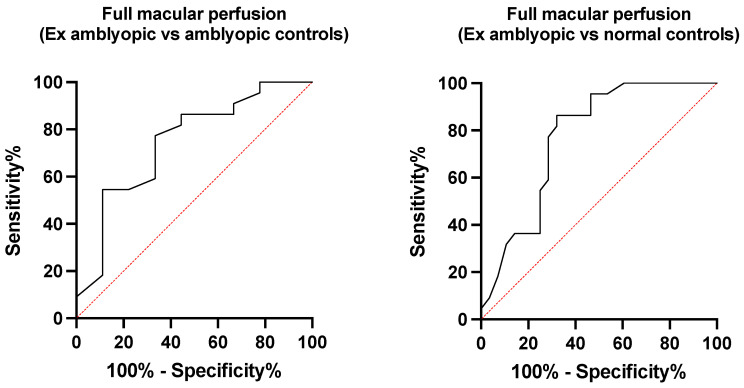
ROC curves showing performances of OCTA macular perfusion in differentiating ex amblyopic eyes from amblyopic eyes (AUC = 0.74) and normal eyes (AUC = 0.77).

**Figure 4:**
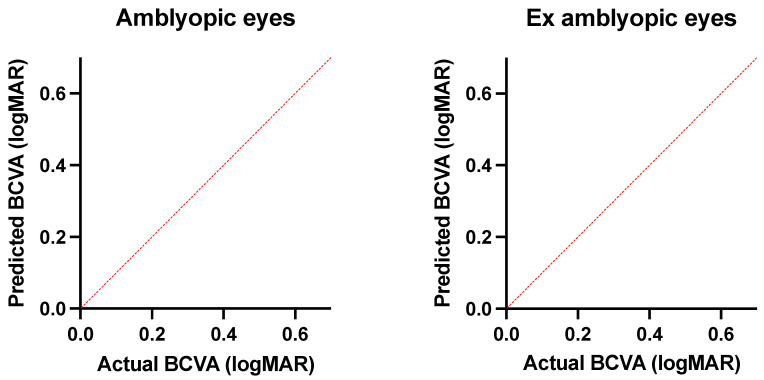
BCVA prediction model based on multiple linear regression of statistically significant OCTA variables in the study groups. The stronger the performance of the model, the closer the dots are to the bisector line. Both the proposed prediction models showed not only statistical significance (*p* = 0.038 in amblyopic patients and *p* = 0.0095 in ex amblyopic patients) but also a good performance (R^2^ = 0.94 for amblyopic patients and R^2^ = 0.68 for ex amblyopic patients).

A total of 59 eyes of 59 pediatric patients were enrolled, among which 28 were normal eyes, 16 were amblyopic eyes (7 strabismus, 5 anisometropia, 4 meridional related amblyopia), and 15 were previously amblyopic eyes (7 strabismus, 6 anisometropia, 3 meridional related amblyopia). No significant differences in age (*p* = 0.07) and sex (*p* = 0.431) were detected among groups. A comparison of spherical equivalent between the three groups revealed a significant difference (*p* = 0.048). Nevertheless, a post-hoc analysis revealed a significant difference in spherical equivalent between normal subjects and amblyopic subjects and normal subjects and ex amblyopic subjects, while amblyopic patients and ex amblyopic patients didn’t differ significantly. The mean duration of treatment in the group that underwent deprivation with a patch (amblyopic and previously amblyopic eyes) was 13.4 ± 1.6 month and no significant difference was detected between the eyes that responded and those that did not. Bland–Altman analysis of FAZ area revealed a good agreement between graders (Bias = 1.27, CI = 0.95–1.46, LA = 29.1%). Results of the two-way MANOVA show how there was a statistically significant difference between the three groups on the combined quantitative dependent variables (*F* (9, 49) = 2.350, *p* = 0.027; Wilks’ Λ =0.432). In particular, a significant difference in the final BCVA (*p* = 0.036) was detected between amblyopic and previously amblyopic eyes (0.35 ± 0.23 and 0.04 ± 0.12 logMAR, respectively). Concerning OCT angiography, the mean outer macular vessel density in the previously amblyopic group was 19.15 ± 0.51%. This was statistically significantly higher than that in both the amblyopic group (18.70 ± 1.14%) and the normal controls (18.18 ± 1.40%) (*p* = 0.014). The previously amblyopic group also significantly differed from both normal controls and amblyopic eyes with regards to the inner (*p* = 0.011), outer (*p* = 0.006), and full (*p* = 0.003) macular perfusion. Moreover, a statistically significant difference in full macular vessel density was noted between previously amblyopic eyes and normal controls (*p* = 0.042) (Table 1, Figure 2). Among OCTA parameters, full macular perfusion displayed the best performance in distinguishing ex amblyopic eyes from both amblyopic eyes (AUC = 0.74) and normal eyes (AUC = 0.77) (Figure 3). A value > 46.85% showed a sensitivity of 59.5% and a specificity of 88.9% in differentiating between ex amblyopic and amblyopic eyes (likelihood ratio = 4.91) while a value > 46.15% efficiently distinguished ex amblyopic eyes from normal eyes (sensitivity = 77.3%, specificity = 71.4%, likelihood ratio = 2.7). Full, inner, and outer macular perfusion and full and outer macular density were evaluated for prediction of BCVA in amblyopic and ex amblyopic group using multiple linear regression (Figure 4). These variables statistically significantly predicted BCVA in amblyopic patients (*p* = 0.038, R^2^ = 0.94), but only inner macular perfusion (*p* = 0.028, B = −0.21) and full macular perfusion (*p* = 0.041, B = −0.12) added statistical significance to the model. The same set of variables statistically significantly predicted BCVA in ex amblyopic patients too (*p* = 0.009, R^2^ = 0.68), with full macular perfusion (*p* = 0.047, B = 0.18) and outer macular density (*p* = 0.01, B = −0.28) adding statistical significance to the model.

## 5. Discussion

Amblyopia is a form of cortical visual impairment and is defined as a condition of unilateral or bilateral decrease in visual acuity that cannot be attributed to structural abnormalities of the eye [5]. Indeed, although alterations in the visual pathways and the visual cortex have been documented, retinal involvement has remained controversial [18,19]. Recently introduced advanced imaging techniques such as OCT and OCTA provide promising additional information on amblyopic retinal structure modifications [20]. Nevertheless, to date, data regarding retinal modifications related to the clinical course of this disease have been sparse. In particular, OCTA characteristics of pediatric patients diagnosed with amblyopia and experiencing a complete post-treatment recovery of their visual function are largely unknown.

To the best of our knowledge, this is the first among the few studies to analyze these features and to perform a comparison with normal and unresponsive amblyopic eyes.

### 5.1. Vascular Density and Perfusion

According to our results, total macular SCP VD and VP in previously amblyopic child patients were significantly higher than those of unresponsive amblyopic and normal eyes, even after adjustment for age, sex, and refractive error. Recently, Zhang et al. [21] compared OCTA findings of patients with untreated anisometropic amblyopia, normal patients, and patients who recovered from the same condition via medical treatment. They detected significantly lower SCP and DCP VD in untreated amblyopic patients compared to both recovered amblyopia and normal patients, with no differences in spherical equivalent between treated and untreated amblyopic groups.

Some considerations could explain the differences in these findings between Zhang’s study and ours. First of all, even if both studies comprised a group of fully recovered amblyopic patients, our study compared it to a cohort of equally treated amblyopic patients whose response to treatment was insufficient to the restoration of VA while Zhang et al. compared fully recovered patients to a group of untreated amblyopic patients. Thus, the results could focus on different aspects of the disease and its clinical course. Furthermore, Zhang et al. did not broach the duration of treatment and VA prior to treatment in recovered amblyopic group. This does not allow for a thorough evaluation of the severity of the amblyopia in the treated group. Most relevantly, our study focused on two groups of patients with severe amblyopia, showing similar VA prior to treatment and undergoing therapy of comparable duration, but reaching different clinical outcomes.

Some other studies focusing on OCT angiographic pattern of amblyopic eyes reported lower macular VD in both SCP and DCP compared to normal and fellow eyes [22,23,24]. Chen et al. [25] evaluated retinal microvasculature in amblyopic children and correlated it with retinal thickness. They found significantly low VD in SCP and a positive correlation between VD and retinal thickness with a greatest difference in anisometropic amblyopia subgroup.

Sobral et al. [26] reported decreased SCP perfusion in amblyopic eyes compared to non-amblyopic controls. In contrast to this, the post-hoc analysis from our study revealed no difference in VD and perfusion between amblyopic and normal eyes. These results are in line with those found by other authors as well [27,28,29].

### 5.2. Retinal Thickness

Huynh et al. [30] proposed that, as a result of the arrest of the normal retinal maturation, which involves movement of the Henle’s fibers away from the foveola, there would be an increased foveal thickness in amblyopic eyes that could be detected on OCT. Different studies have evaluated foveal thickness and macular thickness in patients with different types of amblyopia. Yet, no consensus has been reached on this topic [20]. Borrelli et al. [31] investigated retinal thickness and choriocapillaris (CC) vessel density. They found that amblyopic eyes had an increased CC vessel density that correlated with increased outer parafoveal macular thickness. They speculated that an increased CC VD may be related to the nourishing and thermoregulating role the choroid plays for the retina. So, they hypothesized that a thicker retina might need more blood supply from the choroid. However, we did not find differences in terms of retinal thickness between the three groups. Therefore, we speculate that the increased vascular density found in the amblyopic eyes at the end of the therapeutic period is not related to a structural reason but rather to a functional one.

### 5.3. FAZ

The FAZ area did not significantly differ among the three study groups. Numerous previous studies agree with this finding as they did not find any difference in FAZ measurements between amblyopic and fellow normal eyes, regardless of the findings concerning VD in the macular plexuses [23,24,27,28,32]. Still, a recent study form Araki et al. [29] reported a reduced SCP FAZ area in eyes with unilateral amblyopia due to anisometropia with or without strabismus when compared to the fellow eyes. In addition, enlargement of DCP FAZ in amblyopic eyes was also detected by Sobral et al. [26]. Similarly, Derelican et al. [33] found a wider FAZ in amblyopic eyes of adult patients compared to the companion eye and healthy control eyes. Thus, there does not seem to be enough consensus in order for us to draw some definitive conclusions on the role of the FAZ area in amblyopic eyes. Similarly, even though the FAZ circularity did not significantly differ among the three study groups in our study, Wong et al. found a reduced FAZ circularity in amblyopic eyes compared to the healthy control [34].

Interestingly, in our study, BCVA of both amblyopic and previously amblyopic patients was linearly correlated to SCP perfusion. With regards to this endpoint, there does not seem to be enough evidence from the currently available literature in order to make some definitive statements. A previous paper by Yilmaz et al. found that SCP and DCP were not affected by the severity of amblyopia [32].

Taken together, our findings suggest a compensatory vascular response in amblyopic patients that experience visual restoration after treatment. According to this theory, at birth, both normal and amblyopic eyes are characterized by a similar potential in macular VD and VP. Thereafter, sensory deprivation due to refractive errors or strabismus would lead amblyopic eyes to a developmental arrest and to VA loss. Still, anti-amblyopic therapy seems then to be able to generate a response in the retinal tissue of certain patients that is capable of restoring the visual potential. In a longitudinal study by Pang et al., the authors demonstrated that an arrest in postnatal maturation can result in thicker foveal retinas [35]. They followed patients during anti-amblyopic therapy and found that appropriate therapy contributes to the development of the concave shape of the fovea. Particularly, they demonstrated that the foveal concavity had become deeper after the treatment. We suppose that a compensatory rescue mechanism or an epiphenomenon of retinal remodulation in the form of enhanced vascular development could play an important role in this tissue response to the treatment. A large body of evidence suggests that dopamine functions as a retinal neurotransmitter and/or neuromodulator, influencing receptive-field properties of retinal neurons, gap junctions between horizontal cells, light-adaptive movement of rod and cone photoreceptors, and membrane turnover of rod outer segments [36]. Most of the results of studies on retinas of vertebrates indicate that dopamine synthesis, release, and metabolism are increased by photic stimulation or light adaptation [37]. Since animal models have shown that light deprivation significantly decreases the normal postnatal increase in the dopamine levels of the retina, it is plausible that light influences the development of the retinal dopaminergic system [38,39]. Recently, amacrine-like cells containing dopamine mediators have been shown to make specialized contacts with the retinal vascular complex of several species, including humans and monkeys [40,41]. While the functional significance of these neuronal associations is not fully understood, they may actually have a role in development and autoregulation of blood flow in the retinal vasculature [42]. The explanation of our findings can be found in the early evaluation of patients exactly at the end of the anti-amblyopic treatment. We speculate that the timing of images acquisition can explain why we did not find a statistically significant difference between amblyopic and control eyes and why the previously amblyopic eyes that were more responsive to treatment show the higher vascularity. Moreover, although the external retina is mainly nourished by the choroid, according to neurovascular coupling, it has been shown how a light stimulus can modify the vascular parameters also of the superficial plexuses that nourish the internal retina [43,44].

When evaluating our results compared to those present in the literature, one must take into account that some of the latter were obtained from adult patients. In our study, we included only pediatric patients and evaluated them just as they recovered from amblyopia. Furthermore, some of the differences between the results of previously published papers and ours might be explained by the inclusion of different types of amblyopia and by the inclusion of patients with unilateral or bilateral amblyopia. Competition theory plays a role in the abnormalities of the retina and can result in changes in eyes of patients with unilateral amblyopia that are not detectable in patients with bilateral amblyopia.

In conclusion, our study, given its cross-sectional nature, it can only represent the first step towards the understanding of a possible correlation between treatment and retinal vasculature development. We hypothesize that a compensatory rescue mechanism or an epiphenomenon of retinal remodulation in the form of enhanced vascular development could play a role in retina response to the treatment.

The clinical value of this study lies in the possibility of identifying a biomarker of response to anti-amblyopia therapy, thus adding support to currently available markers that are used to modulate therapy on the characteristics of the single patient. Such personalized adjustment of therapy can contribute to increased adherence and compliance by the patient, which are fundamental elements in the success of anti-amblyopic treatment.

### 5.4. Limitations

Further evaluations on larger populations are advisable to validate our data. In our limited sample, we could not compare refractive and strabismic amblyopic patients. In addition, we were not able to perform axial length measurements, although all comparisons were made with refractive error as a covariate. The latter may have actually adequately accounted for axial length evaluation. Considering the cross-sectional nature of the study, we can only hypothesize a possible correlation between light stimulation and retinal vasculature development. A longitudinal study on the modifications of microvascular structures during anti-amblyopic treatment could better clarify the relationships with visual acuity, could establish whether the subnormal retinal vascularity is a consequence or a cause of amblyopia, and could highlight the role of retinal vascularity in the responsiveness to treatment.

## Figures and Tables

**Table 1:** Results of multivariate analysis comparing general and ophthalmic characteristics among groups. As concerns the variables “sex” and “eye”, result refer to Chi^2^ statistics. *p* values displayed on the last column report the output of post hoc analysis of the analyzed correlations. BCVA = best-corrected visual acuity; FAZ = foveal avascular zone, M = male, F = female, R = right, L = Left. Bold values denote statistically significance difference between the groups.

		Total	Ambliopic Eyes	Ex Ambliopic Eyes	Normal Eyes	*p*
N patients		59	16/59 (27.1%)	15/59 (25.4%)	28/59 (47.5%)	
Sex		M = 26/59 (44.1%) F = 33/59 (55.9%)	M = 9/16 (56.3%) F = 7/16 (43.7%)	M = 5/15 (33.3%) F = 10/15 (66.7%)	M = 12/28 (42.9%) F = 16/28 (57.1%)	0.431
Age		8.9 ± 2.3	7.4 ± 1.6	9.3 ± 2.2	9.6 ± 2.3	0.07
Eye		R = 28/59 (47.5%) L = 31/59 (52.5%)	R = 10/16 (62.5%) L = 6/16 (37.5%)	R = 4/15 (26.7%) L = 11/15 (73.3%)	R = 14/28 (50%) L = 14/28 (50%)	0.127
Spherical equivalent (D)		1.37 ± 1.63	2.33 ± 2.15	2.52 ± 1.96	0.35 ± 0.24	**0.048**
Initial BCVA (logMAR)		0.38 ± 0.34	0.40 ± 0.33	0.38 ± 0.27		0.33
Final BCVA (logMAR)		0.30 ± 0.19	0.35 ± 0.23	0.04 ± 0.12	0.02 ± 0.01	**0.036**
Duration of treatment (months)		13.43 ± 1.61	13.27 ± 1.21	13.64 ± 2.42		0.86
Central macular thickness		276.3 ± 27.2	269.6 ± 28.4	277.8 ± 21.5	279.1 ± 24.2	0.89
FAZ area (mm^2^)		0.22 ± 0.12	0.19 ± 0.13	0.25 ± 0.13	0.23 ± 0.10	0.312
Density	Central	10.8 ± 3.25	11.84 ± 3.55	11.47 ± 2.82	9.95 ± 3.01	0.112
	Inner	18.1 ± 1.32	18.29 ± 1.37	18.50 ± 0.78	17.75 ± 1.45	0.154
	Outer	18.6 ± 1.26	18.70 ± 1.14	19.15 ± 0.51	18.18 ± 1.40	**0.014**
	Full	18.3 ± 1.25	18.54 ± 0.96	18.67 ± 0.83	17.86 ± 1.38	**0.042**
Perfusion (%)	Central	24.6 ± 7.72	27.24 ± 8.42	26.21 ± 7.13	22.36 ± 7.16	0.083
	Inner	43.4 ± 3.46	43.11 ± 3.51	45.07 ± 2.03	42.26 ± 3.69	**0.011**
	Outer	46.2 ± 3.12	46.33 ± 2.53	47.74 ± 0.87	44.97 ± 3.82	**0.006**
	Full	45.0 ± 3.18	44.96 ± 2.71	46.65 ± 1.05	43.73 ± 3.73	**0.003**
